# 5th Congress of the Brazilian Biotechnology Society: Frontiers in Biology

**DOI:** 10.1186/1753-6561-8-S4-I1

**Published:** 2014-10-01

**Authors:** Luiz Antônio Barreto de Castro, Dario Grattapaglia

**Affiliations:** 1 Brazilian Society for Biotechnology, Brasília, Brazil; 2 Embrapa Genetic Resources and Biotechnology, Parque Estação Biológica, PqEB, Av. W5 Norte, Brasília, DF, 70770-917, Brazil; 3 Graduate Program in Genomic Sciences and Biotechnology, Universidade Católica de Brasília - SGAN 916 modulo B, Brasília 70790-160 DF, Brazil

## 

Despite Brazil's increasing production of scientific articles in the last three decades, this growth has yet to translate into an effective contribution to the economic development of this large and diverse country and to the improvement of the life standards of its population. The advances in biotechnology, whose full development relies on the elucidation of the main tenets of biology, plays an increasingly crucial role in food security, environmental remediation, sustainable bio-based industry and public health.

Recognizing the key relevance of biotechnology to the overall development of our country, the Brazilian Biotechnology Society was founded in 1988. The SBBIOTEC was established to promote the overall progress of biotechnology by integrating biosciences, technology development and capacity building, aiming at contributing with positive impacts on the economic development and well-being of the Brazilian society. Scientific in its nature, from 1998 on SBBIOTEC has held Congresses in São Paulo, Salvador, Fortaleza and Santos. For the 5th edition of the SBBIOTEC Congress, held in the beautiful city of Florianópolis, the organizing committee thought it would generate a permanent memory of the content of the event by publishing a BMC Proceedings supplement. By capturing the current status of research and applications of biotechnology in Brazil, SBBIOTEC hopes to provide better visibility and value of the scientific contributions presented by the participants and make such an initiative an everlasting one for future Congress editions.

During five days, in eight scientific sessions, an outstanding team of scientists both from public and private organizations shared their results and visions on the present and future of this fast moving area of science and technology with an audience of over 600 attendees. Some of the current advances of biotechnology research and applications in Brazil were presented together with keynote contributions from international scientists. In total, 47 oral lectures and 264 poster presentations, these last ones mostly by undergraduate and graduate students, were delivered, totaling 311 papers now available online as extended abstracts into this BMC Proceedings supplement. Presentations covered four different areas, namely: agricultural, human health, animal and industrial biotechnology. Besides the Brazilian delegates, scientists from biotech companies in Canada, USA, and Europe also came to the Congress to discuss important issues such as industrial scaling up, animal and insect gene expressions and markers for the early detection of epithelial cancer, to name a few.

The program included a specific section on biofuels biotechnology, a challenging area that can be significantly boosted by metabolic and genomic engineering strategies applied to industrial yeast strains to improve bioethanol production. In human health, stem cells, tuberculosis and cancer were thoroughly discussed with emphasis on monoclonal antibodies in the therapeutic scenario. Pre-clinical and clinical trials were dealt as a critical issue to this process, particularly in light of a recent activist attack that destroyed one of the very few skilled institutions performing pre clinical trials in Brazil. In agricultural biotechnology, an area where Brazil is currently a worldwide key player, a number of important advances were presented in the area of nitrogen fixation in grasses, host pathogen interactions, and vaccine production in plants. Biological control of plant pests and human diseases such as dengue fever, and advances in RNA interference and intragenics were also hot topics of discussion. In animal husbandry the potential production of heparin in Brazil and several advances in animal gene expression were thoroughly discussed. Finally, specific sessions were devoted to the discussion of the legal framework for bioscience development, graduate training and initiatives to enhance biotechnology collaborative networks in Brazil.

In closing this introductory statement, a major acknowledgement is due to the outstanding financial support provided by the Federal District Research Foundation (Fundação de Amparo à Pesquisa do Distrito Federal FAP-DF) that made the Congress and this BMC Supplement possible. Besides the financial sponsorship of FAP-DF and an active Scientific Committee involved in abstract review, a number of people were involved in the organization and logistics. The conference would not have been possible without the valuable contributions of all these players. Given the rewarding feedback received after the Congress, we believe that the goal of providing a good mix of science and social interactions was truly accomplished, which we trust should be well illustrated by the abstracts that now follow these brief opening words.

